# Breast Health Education as a Motivator for Breast Self-Examination Practice in High-Risk Women: Grounded Theory Analysis

**DOI:** 10.2196/83520

**Published:** 2026-01-13

**Authors:** Sumaira Naz, Sureeporn Thanasilp, Wasinee Wisesrith

**Affiliations:** 1 Faculty of Nursing, Chulalongkorn University, 254 Phaya Thai Rd, Wang Mai, Pathum Wan, Bangkok, 10330, Thailand, 66 22181155

**Keywords:** breast self-examination practice, breast health education, breast cancer, high-risk women, breast self-examination

## Abstract

**Background:**

Women in low-resource regions face a higher risk of breast cancer. Implementing a breast health initiative that promotes breast self-examination practice could aid in the early detection and prevention of breast cancer complications.

**Objective:**

This study aimed to explore and comprehend the experiences of high-risk women, focusing on their breast self-examination practice and the factors that influence their effectiveness in managing breast health.

**Methods:**

This research used a qualitative approach to perform semistructured interviews with 11 high-risk women who had a family history of breast cancer recruited from the oncology department of a hospital using purposive and theoretical sampling during the period from August 2024 to April 2025. The analysis of the data was conducted using the grounded theory approach by Strauss and Corbin to formulate a theoretical model for breast self-examination practices.

**Results:**

This study highlighted breast health education as a motivator of and the core category for breast self-examination practice. This study found perceptual, attitudinal, and familial support drivers of breast self-examination practice for early diagnosis of breast cancer and better living.

**Conclusions:**

This study enhances the body of knowledge regarding the experiences of high-risk women. Health care providers play a significant role in using this framework to steer innovative educational interventions that promote breast health in culture-bound communities.

## Introduction

Worldwide, 2.3 million women were diagnosed and 760,000 died of breast cancer (BC) in 2022 [1]. BC is the second most common type of cancer and among the major causes of pathological complications [2]. Approximately 15% to 20% of women diagnosed with BC have a family member who has also been diagnosed with the disease [3]. A family history of BC elevates the likelihood of developing the illness, particularly among close blood relatives who have had BC. Women with a first-degree relative (sister, daughter, or mother) diagnosed with BC face nearly double the risk. If a woman has 2 first-degree relatives diagnosed with BC, her risk increases by roughly 3 times [4].

In Asia, Pakistan records the highest rate of BC incidence at 23.1% [5]. Additionally, 23.8% of women in Pakistan have a family history of BC. The average age of family members diagnosed with BC has been found to be 49.2 years [6]. Moreover, 95.2% of these individuals have at least one family member who was affected. The most common relative diagnosed with BC is the mother, accounting for 47.6% [7]. The World Health Organization–recognized tools for BC screening are mammography, clinical breast examination, and breast self-examination (BSE) [1]. Although mammography has proven to be a reliable and valid BC screening method, awareness of this tool and its accessibility and affordability to women have been low in poor-resource countries [8]. BSE and clinical breast examination come in handy in such countries. BSE practice is a more acceptable method due to cultural issues, and evidence has proved that 40% of diagnosed BCs are detected through BSE [9], thus validating the usefulness of the procedure in BC screening [3].

Among high-risk Pakistani women with a family history of BC, merely 15% are knowledgeable about the disease, and only 4.18% use BSE as a screening measure for BC [10]. Just 1% regularly practices BSE, whereas 3.6% do so occasionally [11]. Furthermore, late-stage presentation of BC (stages III or IV) is prevalent throughout the country, with nearly 35.2% of delayed cases occurring among high-risk Pakistani women [12]. It has been demonstrated that 40% of diagnosed BCs are identified through BSE [13].

In Pakistani culture, the concept of the “breast” is more associated with sexuality than with health, making discussions about it taboo due to conservative societal norms [14]. Cultural influences significantly affect breast health awareness among Pakistani women, with many refraining from performing BSE due to the stigma surrounding self-examination and embarrassment over discussing private body parts or undergoing medical assessment [15,16]. Misunderstandings, societal expectations, and false beliefs hinder the BSE practice and lead to delays in seeking help among women. The influence of culture has resulted in women not being motivated to carry out BSE or being taught how to [17].

Despite the significance of cultural values, there is a lack of research on the implementation of BSE practice measures among women at high risk. To improve this situation, it is essential to investigate the BSE practice viewpoints of high-risk women. This study used a grounded theory approach to create a conceptual understanding based on participants’ lived experiences, aiming to formulate a conceptual model or theory rooted in participants’ perspectives.

The purpose of this study was to explore and comprehend the experiences of high-risk women, focusing on their BSE practice and the factors that influence their effectiveness in managing breast health.

## Methods

### Study Design

This study used the grounded theory approach by Strauss and Corbin [18] and adhered to the COREQ (Consolidated Criteria for Reporting Qualitative Research) checklist to ensure rigor [19]. Grounded theory is a qualitative research method aimed at developing a theory that is firmly based on data that are collected and analyzed systematically. This approach is especially effective for examining intricate social processes, such as BSE practice, as it seeks to understand how people develop and sustain behaviors about a specific health issue.

### Participant Selection

The data were gathered between August 2024 and April 2025. The researchers applied both purposive and theoretical sampling to select data sources and participants. Oncology nurses asked 11 high-risk women to participate in the study, and no one declined to take part in the interviews. The timing and date were arranged based on the availability of the participants, and the researchers provided thorough explanations about the study. At first, purposive sampling was used to identify participants who met certain eligibility criteria: (1) female participants with mothers diagnosed with BC, (2) proficiency in the Urdu language, and (3) willingness to take part. When the investigation advanced, theoretical sampling was used to enhance evolving theory. Theoretical sampling is an iterative process in which data collection and analysis are conducted simultaneously, using the emerging analysis to guide the selection of subsequent data to collect. Collected data were coded and analyzed to form initial concepts, categories, and themes. The selection of new participants was based on their marital status (unmarried, married, or widowed) and level of education (primary school, middle school, tenth grade, or higher) to understand the concepts, fill the gaps, refine categories, and expand the theory. Theoretical sampling persisted until data saturation was reached, when no further relevant data or insights were produced. The aforementioned 11 high-risk participants from diverse communities took part in the study (Table 1).

**Table 1.** Participant characteristics.

Participant ID	Age (y)	Marital status	Educational level	Region
HRW1^a^	21	Married	Tenth grade	Urban
HRW2	24	Married	Tenth grade	Urban
HRW3	22	Married	Primary school	Urban
HRW4	21	Unmarried	Tenth grade	Rural
HRW5	26	Married	Graduation (16 years of education)	Urban
HRW6	24	Married	Primary school	Rural
HRW7	22	Married	Primary school	Rural
HRW8	27	Married	Tenth grade	Urban
HRW9	24	Widowed	Primary school	Semiurban
HRW10	25	Married	Middle school	Rural
HRW11	23	Unmarried	Twelfth grade	Semiurban

a HRW: high-risk women.

### Ethical Considerations

The Research Ethics Committee of the Institute of Allied Health Sciences associated with the hospital provided ethics approval for this study, with reference IAHS/WMC/786/008-02. All participants provided informed consent, and information was provided on their right to leave the study at any point without facing repercussions. Participant data were anonymized to ensure confidentiality, and all research materials were securely stored. Participants were not provided any compensation for their participation.

### Data Collection

Data were gathered through semistructured interviews (Table 2). These interviews were conducted in person, with audio recordings made of participants, along with observations and verbatim transcriptions. Each interview lasted approximately 35 to 50 minutes. Field notes were kept providing contextual details. Several participants were interviewed more than once, with 81.8% (9/11) taking part in a second interview to enhance the data and facilitate clarification, deeper exploration, and a richer understanding of emerging themes. Data were gathered until data saturation was achieved, which is defined as the point at which no new themes or insights arise. Due to logistical constraints and participant preferences, transcripts were not returned to participants for member verification. Nonetheless, to maintain ethical standards and ensure the accuracy of the transcriptions, a method of double transcription and validation was used [20,21].

**Table 2.** Interview guideline.

Category	Questions
Opening questions	“Do you think that you should take care of your breast health? Or do you think that breast health is important for women health?” “What do you know about breast health as you are at risk of breast cancer or what do you know about breast cancer as your mother is suffering from the same condition?” “Do you know how to do breast self-examinations practice or what are the methods of breast self-examination practice?” “Do you examine your breast? Or do you think that you should do breast self-examination practice?”
Probing questions	“Do you have the ability or confidence to do your breast self-examination practice?” “What changes do you observe during breast examination?” “Can you explain the change or discuss the changes with anyone, or do you think that change should be discussed?” “What will you do to manage changes in your breasts to maintain your breast health?” “Do you think that you need support or assistance, and how did you receive such support?”
Closing question	“Is there anything you would like to share or add?”

### Analysis

**Figure 1.**
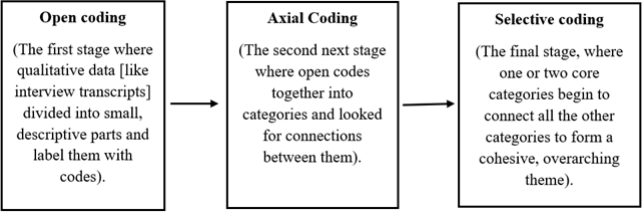
Coding process.

Analysis of the data was conducted following the grounded theory approach by Strauss and Corbin [18], which involved open, axial, and selective coding (Figure 1).

**Figure 2.**
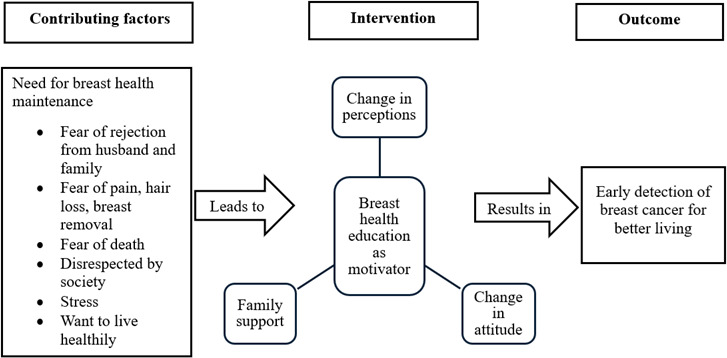
The theory of breast health education as a motivator among high-risk women for breast self-examination practice.

Coding of interview transcripts was done using verbatim data. Conceptual sensitivity to achieve reflexivity and analytic consensus was maintained by using multiple coders in discussion, where codes were reached through ongoing discussions and adjustments to the coding framework. The coding tree (Table 3) shows that the initial codes were organized into 5 subcategories under a single main category and were subsequently combined to create the final thematic model. Constant comparative analysis was used for the development of themes, in which new data were continually assessed against existing codes and categories. This approach allowed for the refinement of the emerging theory and affirmed its applicability across diverse participant experiences [22,23], directed at identifying repeated patterns and connections within the data [20,24,25]. The process resulted in the documentation of the key themes: the desire for breast health awareness, family support, change in perceptions and attitude, and early diagnosis of BC (Figure 2).

**Table 3.** Coding process for the category of breast health education as a motivator.

Level of coding	Code or category	Participant code
Open coding	Realization of health issues related to BC^a^. Awareness of BC symptoms. Motivation to seek information about BC. Cultural issues related to delay in health-seeking behavior. Family support from mother Change of perceptions Breast health awareness Change in attitude	“I know that I AM at risk of breast cancer because my mother is suffering from this and I saw my mother’s condition.” [Participant 1] “My mother discusses her problems with me, and I feel her pain.” [Participant 5] “My mother is suffering from pain, stress, hair loss, and many other problems.” [Participant 3] “My mother told me that my condition was better if she know about breast cancer and she wish for me to have all information about breast cancer, and I also want to know about this.” [Participants HRW 5] “My mother hides her problem from all family and when she can’t bear her problem than she disclosed to her mother-in-law and she agreed to visit the doctor.” [Participants HRW 7] “I saw my mother’s condition, and I was worried. My mother guided me to touch the breast is not wrong it’s our body part; we can discuss our problems with lady doctor because she is also women like us. She can understand us.” [Participants HRW 4] “I did breast examinations to feel any change in my breast like pain, swelling for early identification of cancer.” [Participants HRW 11]
Axial coding	Category: BSE^b^ practice (subcategory 1: breast health maintenance; subcategory 2: symptoms awareness) Category: influential factors (subcategory 1: change in perception of BC and BSE; subcategory 2: change in attitude; subcategory 3: family support)	Open codes: “I can do breast examination by touch and observation.” [Participant HRW 4] Open code: “I know the symptoms like pain in breast, swelling in breast, discharge.” [Participant HRW 6] Open code: “My mother told me that breasts are like other body parts, breast problems are like other problems of body, breast touch is not wrong.” [Participant HRW 9] Open code: “Realizing the importance of early diagnosis, performing Breast examination.” [Participant HRW 11] Open code: “I can understand breast health issues and have support from mothers in early health seeking behavior such as to visit doctor for my problem.” [Participant HRW 9]
Selective coding	Category: breast health education as motivator	Realization of breast health issues, and need for BSE practice for identification of any breast change, and that leads to early BC diagnosis for healthy living

a BC: breast cancer.

b BSE: breast self-examination.

### Credibility and Trustworthiness

The reliability and credibility of this study were maintained through implementation of several approaches as suggested by recognized qualitative research guidelines [26]. These included (1) member checking, in which initial interpretations and findings were shared with high-risk participants to check the accuracy and authenticity; (2) triangulation, which involved comparing various data sources—such as interview transcripts and field notes—to enhance the validity of the results; (3) investigator triangulation through ongoing team discussions during the data analysis phase to confirm consistency and individual bias reduction; and (4) an audit trail by keeping thorough documentation, such as coding decisions, to facilitate external review and transparency.

## Results

The core category of the study was breast health education as a motivator, emphasizing the vital role that awareness and understanding of one’s breast health plays in promoting BSE practice for BC screening (Figure 2).

### Contributing Conditions: Need for Breast Health Maintenance

Understanding the mother’s BC situation and being aware of BC symptoms such as pain or hair loss, fear of rejection from society, and stress creates the need for breast health maintenance and can inspire participants to take part in screening activities such as BSEs for early detection and instill hope for improved health. Family support, especially support from mothers who realized their own situation and understood their daughters’ need for breast health, motivated participants to change their perceptions of health care choices and the way in which they sought health services and build a positive attitude to engage in health-seeking behaviors such as BSE practice.

### Intervention Conditions: Key Factors Influencing BSE Practice

#### Change in Perceptions

Cultural factors significantly influence health care choices and the way in which individuals seek health services. The participants recognized that being aware of breast health is a key aspect of women’s health. They adjusted their views regarding breast health and, through family support, appeared motivated to pursue health-seeking behaviors.

#### Change in Attitude

Cultural values shape women’s attitudes, leading to feelings of anxiety surrounding BC diagnoses and embarrassment when discussing the topic. Participants acknowledged the significance of early detection for improving quality of life and demonstrated motivation for maintaining breast health.

#### Family Support

In Pakistani culture, family support, particularly from mothers, plays an essential role in influencing their daughters’ perceptions regarding health care choices. The encouragement from mothers has a significant impact on promoting their daughters’ health.

### Outcome: Early Detection of BC for Better Living

The objective of BSE practice is to facilitate the early identification of BC through health education, thereby enhancing quality of life and promoting better living. Early diagnosis of BC significantly increases the likelihood of survival for patients.

This core category emerged in various driving themes, such as the realization of breast health issues, the change in participants’ perceptions and attitude, and participants’ need for BSE practice for identification of any breast change that would lead to early BC diagnosis (Table 4).

**Table 4.** Major themes with participant quotes.

Theme	Participant quote
Need for breast health maintenance	“I am aware that I have a higher risk of developing breast cancer because my mother is currently facing this illness, and I have witnessed her suffering from pain, stress, hair loss, and various other challenges.” [Participant HRW 3]
Change in perceptions	“I observed my mother’s situation, and it concerned me. My mother taught me that examining our breasts is natural; it’s a part of our bodies, and we can talk about our issues with a female doctor because she is also a woman like us. She can realize our experiences.” [Participant HRW 4]
Change in attitude	“I possess knowledge about breast self-examination and various methods, like using my fingers to feel any changes in the breast, such as discomfort or swelling, and I can also monitor any change between both breasts.” [Participant HRW 11]
Family support	“My mother shares her issues with me; I empathize with her struggles. She mentioned that her situation could have improved if she had been informed about breast cancer, and she hopes I have all the knowledge regarding it, which I also wish to acquire.” [Participant HRW 5] “My mother reminds my sister and me to conduct monthly breast checks, always assuring us that she is by our side.” [Participant HRW 9]
Early detection of breast cancer for better living	“I perform breast examinations every month because I am aware of my mother’s situation; she was diagnosed very late. The doctors informed us that if she had been diagnosed sooner, her condition would be in a better state than it is now. Therefore, I have hope that I will not endure the same fate as my mother. I am determined to lead a healthier life.” [Participant HRW 1] “I perform breast self-examination regularly with a hope that I will live a healthy life.” [Participant HRW 8]

## Discussion

### Principal Findings

The results of this research highlight the importance of health education in encouraging BSE practice among Pakistani high-risk women. This research adds to the grounded theory concerning BSE practice by demonstrating how health education influences various elements of preventive and promotional health care, such as perceptual change, attitudinal change, and familial support for BSE. The primary theme, breast health education as a motivator, emerged as a key factor influencing behaviors related to BSE practice. This observation is consistent with the health belief model, which suggests that people are likely to engage in health-promoting behavior when they recognize a significant degree of susceptibility to a health issue [27]. The findings of this study on BC symptoms served as a trigger for embracing BSE practice, underpinning the idea that awareness of potential health risks fosters change in behavior [28].

This research emphasizes that educating individuals about breast health plays a critical role in encouraging them to perform BSE. Those with greater knowledge about BC were found to be more motivated to engage in practices that promote breast health. This highlights the significance of educational programs aimed at improving people’s understanding of BC, the misperceptions about it and its associated risks, and the advantages of different screening strategies [29].

Knowledge affects an individual’s views and interpretations of sociocultural contexts, as well as their ability to foresee results and make choices. Greater awareness and a favorable change in perceptions of BC and the taboos of BSEs can greatly enhance BSE practice [30]. This aligns with the principles of social cognitive theory. According to social cognitive theory, knowledge affects perception by influencing how people decode social situations and anticipate outcomes [31].

Education about breast health can alleviate feelings of embarrassment and fear, resulting in a more favorable outlook on BSEs and the pursuit of medical consultations due to cultural impact [32]. These conclusions correspond with the change theory and the stages of change model by Lewin [33], which can be used to foster more constructive attitudes toward change. Individuals and groups adjust to new circumstances, handle resistance, and reinforce new behaviors [33].

Cultural context affects behavior change. The PEN-3 cultural model has also already demonstrated how cultural context matters in interventions, such as those for cancer awareness and screening [34]. Naturally occurring support from family members has been shown to increase healthy lifestyle behaviors such as BC screening measures (eg, BSE) through providing information and role-modeling. Family members have an impact on women’s decisions and actions throughout their BC journey, such as (1) confirming breast changes, (2) managing personal emotions, (3) seeking the information, (4) seeking alternative forms of treatment, and (5) advocating for conventional treatment [35]. Family support, especially from mothers, acts as a significant environmental factor that plays an important role in influencing BSE practice. Respectable family support increases a woman’s awareness of and interest in undergoing early cancer screening. If a woman receives good emotional support, then she is more likely to behave well for her health [36].

The influence of culture on perception, attitude, and family support, especially from mothers, regarding BSE practice is a core observation of this study. The participants’ ability to adopt cultural practices such as family support and change in perception of and attitude toward women’s breast health in response to health needs determines the importance of culturally sensitive interventions [37]. This study highlighted the need for BSE practices that are culturally and contextually relevant, such as breast health education targeting participant negative cultural beliefs related to BC and BSE (eg, the taboo of touching oneself) by fostering positive perceptions of this body part, such as the fact that it is a woman’s body part, which means that it is also a part of health and not only a part of sexuality, and fostering a positive attitude about BC and its screening measures (eg, BSE) by encouraging women to talk about BC with their family members (eg, with their mothers). Family support motivates women regarding the fact that BSE is not a wrong concept. The breast is a part of their body, which they have a responsibility to be aware of. All these efforts will lead toward behavior change and the promotion of preventive behaviors.

### Limitations

This study was carried out at a single hospital, representing a limited group of high-risk women in the area. Grounded theory seeks to create theories based on contexts and data, which might restrict the applicability of the results to different populations.

### Conclusions

This study offers convincing evidence underscoring the vital role of breast health education in promoting BSE practices among high-risk women in Punjab, Pakistan. The findings provide essential insights into how improved breast health education can bring about positive changes in attitudes, perceptions, and the involvement of family support systems. This research marks the first effort to develop a grounded theory that presents a new conceptual model to understand the processes related to effective breast health management in similar culture-bound communities.

## Abbreviations

BC: breast cancer
BSE: breast self-examination
COREQ: Consolidated Criteria for Reporting Qualitative Research
